# A Decision Support System for Economically Sustainable Sheep and Goat Farming

**DOI:** 10.3390/ani10122421

**Published:** 2020-12-17

**Authors:** Sotiria Vouraki, Ioannis Skourtis, Konstantinos Psichos, Wendy Jones, Carol Davis, Marion Johnson, Leticia Riaguas Rupérez, Alexandros Theodoridis, Georgios Arsenos

**Affiliations:** 1 Laboratory of Animal Husbandry, School of Veterinary Medicine, Faculty of Health Sciences, Aristotle University, 54124 Thessaloniki, Greece; arsenosg@vet.auth.gr; 2 Integrated Information Technology and Digital Communication, 11525 Athens, Greece; yannis@integrated.gr (I.S.); dinospsichos@gmail.com (K.P.); 3 The Sheep Centre, National Sheep Association, Malvern, Worcestershire WR13 6PH, UK; wendyjones640@gmail.com or; 4 Agriculture and Horticulture Development Board, Kenilworth, Warwickshire CV8 2TL, UK; Carol.Davis@ahdb.org.uk; 5 Organic Research Centre, Trent Lodge, Stroud Rd, Cirencester GL7 6JN, UK; marion.j@organicresearchcentre.com; 6 Oviaragón Pastores Cooperative Group, 50014 Zaragoza, Spain; lriaguas@oviaragon.com; 7 Laboratory of Animal Production Economics, School of Veterinary Medicine, Faculty of Health Sciences, Aristotle University, 54124 Thessaloniki, Greece; alextheod@vet.auth.gr

**Keywords:** sheep, goat, management, efficiency, production systems, decision support, web application

## Abstract

**Simple Summary:**

The viability of the European sheep and goat sector is threatened by market, policy, social and technical challenges. To address such challenges, innovations for efficient farm management, especially with decision support capabilities, are considered a strategic priority. The iSAGEDSS is a web application, based on the Happy GoatS solution, which allows dairy and meat small ruminant farmers to make annual management plans by testing future what-if scenarios. It is designed for the meat sheep production system in the United Kingdom and Spain, the dairy sheep production system in France and Greece and the dairy goat production system in Greece. Moreover, it addresses all important farm parameters (flock size, production, processing, grazing, feeding, income and costs and farm prices) and utilizing an energy and protein-based algorithm for nutritional management assessment it produces reports, which are focused on profitability and productivity. Environmental-related figures are also estimated. Results are illustrated with simple and easily understood charts and plots. Case study application results showed high prediction accuracy and demonstrated the ability of the system to promote efficient farm management. In this regard, iSAGEDSS is expected to help farmers to adjust to the challenges faced by the sector and remain sustainable.

**Abstract:**

The European sheep and goat sector is characterized by low professionalization and management training. Moreover, it is fragmented in terms of production aims and farming systems. Here, iSAGEDSS, a web-based application allowing dairy and meat small ruminant farmers in different countries to make annual management plans by testing future scenarios, is presented. Data were obtained for the meat sheep (United Kingdom and Spain), dairy sheep (France and Greece) and dairy goat production systems (Greece) from partners of the Innovation for Sustainable Sheep and Goat Production in Europe (iSAGE) project. These were used to set default values and ranges for all important farm parameters in each system and country. An algorithm was developed assessing nutritional management and its impact on production and financial performance. Reports focus on profitability, productivity and environmental sustainability. A case study in three dairy sheep farms in Greece was performed. In each case, an evaluation scenario was created using actual farm data that were compared with the estimated ones. Two scenarios testing management decisions for gross margin maximization and milk pricing fluctuations were created. Application results showed high prediction accuracy for gross margin and production estimation (error of circa 9% and 4%, respectively). Moreover, the ability to promote financial, production and grazing management efficiency was demonstrated.

## 1. Introduction

Market and policy measures have resulted in financial difficulties for sheep and goat farmers. Specifically, the sector is characterized by inadequate farm-gate prices and increased production costs. As a result, many farms operate under the profitability threshold [1,2,3]. To compensate for these financial difficulties, sheep and goat farmers were among the first in the agricultural industry to receive public support from the Common Agricultural Policy [4]. However, such measures have not managed to improve productivity or the overall competitiveness of the sector [3,5]. Moreover, European Union (EU) policies are constantly changing; currently, farmers with land rights are being rewarded, while in the near future, environmental performance will also be taken into account [6]. Such challenges require profound transformations regarding how farms are currently being managed.

Moreover, sheep and goat farming require specific skills and a well-established management plan. However, the sector is lacking in professionalization and management training, resulting in varied levels of productivity [1,3]. At the same time, technology and innovation, which could help to modernize small ruminant farming, have remained relatively stagnant and neglected by both the supply chain actors and mainstream research [7].

Cloud-based decision support systems utilizing predictive modeling have been successfully developed and applied in the field of agriculture, especially for efficient crop management [8,9,10]. In the farm animal industry, emphasis has been placed on assisting dairy cow farmers in decision-making through predictive modeling for animal production and financial performance [11,12]. Regarding small ruminant farm management, there are some available tools that aim to help sheep and goat farmers towards decision-making [13,14,15,16,17,18,19,20,21,22,23]. However, most of the current solutions do not offer a holistic management approach that takes into account all important farm parameters [14,18,21] or they require a great amount of effort for data input via non-user-friendly (complex and/or poorly structured) interfaces [13,15,16,19]. Data input is also sometimes linked to breeding and milk recording schemes undertaken by farmer associations/cooperatives, hence excluding farmers that are not members of such associations [23]. Moreover, they use historic farm data and, therefore, they do not offer decision support capabilities for future planning. Such issues, along with cost-effectiveness and the educational status of farmers, have been reported to tackle the adoption of relevant technologies [24,25,26]. Moreover, the need for reliable farm-level data and analytical models for high-accuracy decision support tools that are of relevance to the users has been highlighted [25,27,28].

In a previous work, we described Happy GoatS (http://happygoats.eu/), a web application, which focuses on facilitating farm management decisions through the projectional analysis of future management data [17]. However, this system was designed for the dairy sheep and goat production systems in Greece based on challenges and issues identified at the time. Given that the European small ruminant sector is fragmented in terms of production aims and that farm types vary between Member States [5,29], our objective was to develop a decision support system for efficient/sustainable farm management that could facilitate sheep and goat farmers of various production systems and countries. Here, iSAGEDSS, a model-driven web application that allows dairy and meat small ruminant farmers in Europe to make annual management planning decisions by testing future what-if scenarios, is described. Sustainable farming is addressed in terms of production, economic resilience and environmental integrity. The latter is defined by grazing management indicators (pasture availability and stocking rate). A case study application from dairy sheep farms in Greece is also presented.

## 2. Materials and Methods

### 2.1. Conceptualization

The iSAGEDSS was developed within the Innovation for Sustainable Sheep and Goat Production in Europe (iSAGE; https://www.isage.eu/) Horizon 2020 project. The idea was based on an existing web application, Happy GoatS, designed and developed by the authors [17]. For the purposes of the iSAGE project and based on key findings regarding challenges and sustainability issues of the small ruminant sector [3], an extension to the above application was designed in order to accommodate the needs of diverse production systems and countries within Europe.

### 2.2. Input Data, Default Values and Acceptable Ranges

Data were obtained from industry and research partners of the iSAGE project for different production systems and countries: the meat sheep production system in United Kingdom (UK) and Spain, the dairy sheep production system in France and Greece and the dairy goat production system in Greece (Table 1).

These data were used to set default values that characterize the management of an average typical farm and acceptable ranges (where applicable) in each production system and country for seven different categories of input farm parameters. Such categories included flock size, production (animal weights and milk, meat and wool production according to the production system), processing (only for the dairy systems), grazing (area, time, distance and available pasture), feeding (forage and concentrate feedstuffs), income and variable costs and prices (for products and feedstuffs) parameters. The initial set of farm parameters (for each production system and country) was presented and discussed with research and industry (farmers and consultants) participants across Europe during regional workshops of the iSAGE project. Industry consultants from the UK pointed out the need to include more options of forage feedstuffs (asides hay, straw and silage) and, specifically, fodder beet and stubble turnips. Therefore, in order to better meet the needs of the UK meat sheep production system, the above feedstuffs were included in the UK model. Based on all the above, five input data forms with different sets of parameters as well as default values and acceptable ranges per production system and country were developed (Supplement S1 Tables S1–S5).

### 2.3. Algorithm

**Figure 1:**
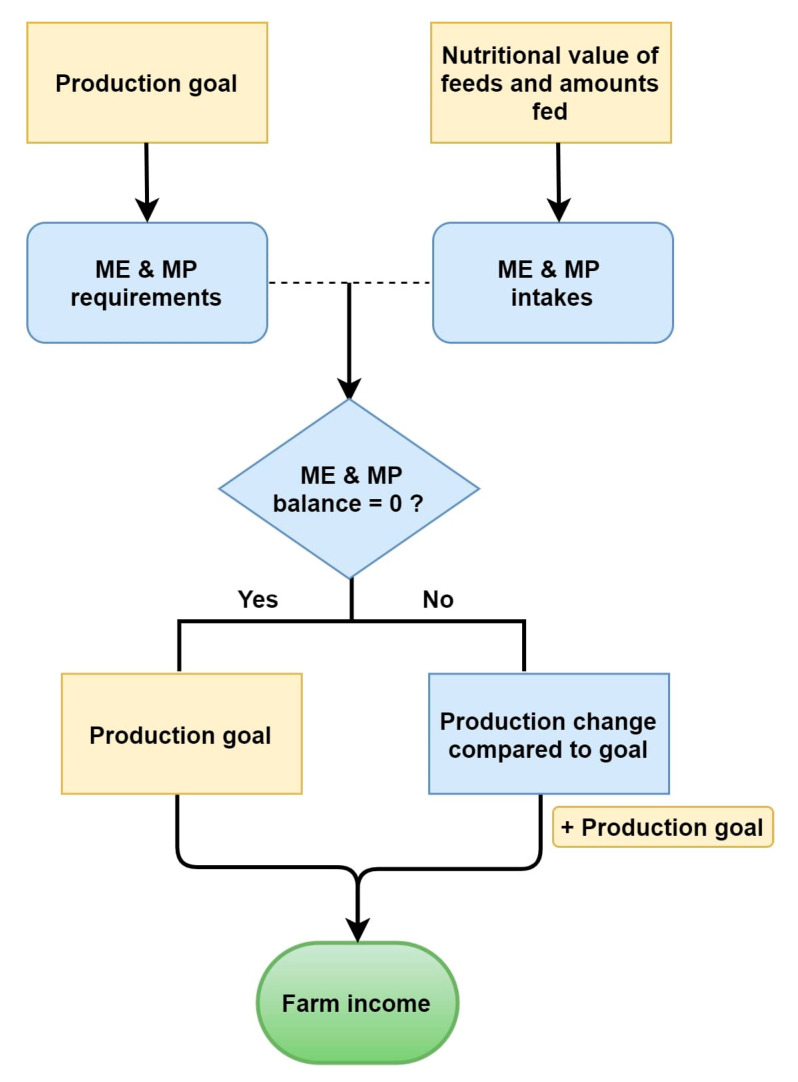
Flow diagram with the basic steps of the iSAGEDSS algorithm; ME and MP refer to metabolizable energy and metabolizable protein, respectively.

An algorithm was developed based on the energy and protein requirements of sheep (lambs, ewes and rams) and goats (kids, does and bucks), according to their production aim and stage, in order to assess nutritional management and its impact on production and financial performance (Figure 1). A part of the algorithm had been developed by the authors for the Happy GoatS solution [17]; however, adjustments and additions were incorporated.

For both dairy and meat production systems, three periods were defined for ewes/does: (i) lactation period, (ii) dry period excluding last month before birth and (iii) last month before birth. Last month before birth was differentiated from the rest of the dry period due to the rapid increase in nutrient demands for the growth of the fetus [30,31]. Moreover, for the meat sheep production system, finishing lambs and replacement lambs were differentiated and their desirable growth estimated using Equations (1) and (2), respectively.

(1) $$G_{r}\;=\;\frac{\left(0.66\;\times \;\mathrm{AW}\right)\;-\;\mathrm{BW}}{\mathrm{AM}}$$

where G_r_ = growth of replacement lambs (kg/day); AW = adult weight (kg); BW = birth weight (kg); AM = age at mating (in days).

(2) $$G_{f}\;=\;\frac{\left(\frac{\mathrm{CW}\;\times \;100}{\mathrm{DP}}\right)\;-\;\mathrm{BW}}{\mathrm{AS}}$$

where G_f_ = growth of finishing lambs (kg/day); CW = carcass weight (kg); DP = dressing out percentage (%); BW = birth weight (kg); AS = age at slaughter (in days).

In all cases, the equations for calculating the nutrient requirements (metabolizable energy and protein) of sheep and goats for maintenance, growth, pregnancy and milk production, where applicable, and dry matter appetite were obtained from the existing literature using the Agriculture and Food Research Council (AFRC) system [30].

The algorithm estimates the average daily metabolizable energy, digestible undegraded true protein and effective rumen degradable protein intake of the different animal categories based on the energy and protein in feeds, respectively, and the estimated dry matter appetite of animals. The microbial crude protein intake is also estimated using Equation (3):

(3) $$\mathrm{MCPI}\;=\;y\;\times \;\left(\mathrm{MEI}\;-\;\left({\mathrm{DMA}\;\times \;\mathrm{ME}}_{\mathrm{fat}}\;\times \;\mathrm{EE}\right)\right)$$

where MCPI = microbial crude protein intake (g/day); y = values of microbial crude protein synthesis (9 g at maintenance, 10 g for growth and 11 g for lactation); MEI = metabolizable energy intake (MJ/day); DMA = dry matter appetite (kgDM/day); ME_fat_ = metabolizable energy from fat (35 MJ/kgDM as suggested by McDonald et al. [31]); EE = ether extract (0.02 kg/kgDM as suggested by McDonald et al. [31]).

Then, the average daily metabolizable protein intake is estimated with Equation (4) based on the suggestions of AFRC [30]:

(4) MPI = min((0.6375 × MCPI) + DUPI, (0.6375 × ERDPI) + DUPI)

where MPI = metabolizable protein intake of each animal category (g/day); MCPI, DUPI and ERDPI = microbial crude protein, digestible undegraded true protein and effective rumen degradable protein intakes (g/day), respectively.

Based on all the above, the algorithm allows for the calculation of energy and protein balance (the difference between metabolizable energy/protein requirements and intakes) for each animal category. The latter provides the basis for estimating production-related figures such as live weight and milk production change of lactating animals in dairy sheep and goat farms or live weight and carcass weight change in meat sheep farms; change refers to the difference between the goal of production and the actual production achieved based on energy and protein balance. These production estimates are then taken into account for the estimation of the farm’s income (Figure 1). Specifically, the estimated values for milk production change and carcass weight change are added to the corresponding production goal for calculating income from milk and meat, respectively. For estimating milk production and live weight change, the directions of AFRC [30] were followed using the appropriate values for each animal category depending on whether the energy and protein balance would be negative or positive. For the estimation of carcass weight change of finishing lambs, Equation (5) was used:

(5) $${\mathrm{CWC}}_{f}\;=\;\frac{\mathrm{LWC}\;\times \;\mathrm{DP}}{100}$$

where CWC_f_ = carcass weight change of finishing lambs (kg); LWC = live weight change of finishing lambs (kg) based on energy and protein balance; DP = dressing out percentage (%).

Additionally, the algorithm allows for estimating pasture availability at the end of the year based on the annual pasture available and the estimated annual total pasture intake of all animals. To estimate the pasture intake of animals, a two-step approach is followed. Initially, a first estimate of the potential daily pasture intake is calculated for each animal category according to their dry matter appetite and the amounts of supplemented feeds they receive. If the first estimate of the annual total pasture eaten by all animals exceeds the amount of pasture available, a second, reduced, estimate of daily pasture intake is calculated for each animal category using Equation (6) according to the directions of the Agriculture and Horticulture Development Board [32]:

(6) $${\mathrm{PA}}_{2}\;=\;\left(\frac{U\;\times \;\left(\mathrm{APA}\;-\;R\right)}{1.1\;\times \;\mathrm{TP}}\right){\;\times \;\mathrm{PA}}_{1}$$

where PA_2_ = second estimate of pasture intake for each animal category (kgDM/day); U = utilization (a maximum of 93% assumed); APA = annual pasture available (kgDM/hectare per year); R = residual (a minimum of 300 kgDM/hectare assumed); TP = estimate of total pasture eaten by all animals (kgDM/hectare per year); PA_1_ = first estimate of pasture intake for each animal category (kgDM/day).

Finally, simple equations were built to estimate useful farm statistics which affect performance, such as ram/buck to ewe/doe ratio, stocking rate, weaning percentage and animals per laborer.

### 2.4. Architecture of the Application

The iSAGEDSS application was architectured around the model-view controller design (MVC) pattern consisting of a RESTful Web Service and a Single Page Application (SPA). The representational state transfer (REST API) is implemented using Java technologies with the following main components: (i) Jersey (Eclipse Foundation, Ottawa, ON, Canada) used as a library for JAX-RS (Java API for RESTful Web Services), (ii) Guice (Google, Mountain View, CA, USA) used as a dependency injection and annotations framework and (iii) Hibernate (Red Hat, Raleigh, NC, USA) used as the application’s object relational mapping (ORM) framework.

The SPA that the end user accesses is an implementation based on the AngularJS (version 1.5.8) framework (Google, Mountain View, California, USA) along with HTML5 (HyperText markup language) and CSS3 (cascading style sheets). The Web Service application and SPA, which in unison offer the iSAGEDSS solution, are being deployed on an Apache Tomcat Web Application Server (version 8.5.6, Apache Software Foundation, Forest Hill, MD, USA).

All the aforementioned servers and components are hosted on a virtualized operating system (OS) environment running CentOS Linux (release 7.7.1908, The CentOS Project and Red Hat, Raleigh, NC, USA). The virtual machine is provided by The DigitalOcean^®^ Cloud (Digital Ocean, New York, NY, USA).

### 2.5. Data Management and Storage

Designated web forms were created for data input. Data are validated against a range of values’ limitations and erroneous input. Specifically, data input is checked for correctness and then compared with theoretical minimum and maximum limits per category imposed by the production system and country. These data are then processed with a model algorithm, which provides results for farm management decisions.

To ensure the protection of farmers’ data, the application implements authentication and authorization processes. Thus, a user must access the system using their unique username and password and they can access only their own farm’s data. All data, both application’s configuration and those submitted by the end users, are stored on a MariaDB relational database management system (RDMS) server (version 5.5.64, MariaDB Foundation, Middletown, DE, USA).

### 2.6. A Case Study Application from Greece

For the purposes of evaluation and demonstration of the utility of the decision support system, a case study application from the dairy sheep production system in Greece is presented. The dairy sheep production system in Greece was chosen on the basis of its lower sustainability scores compared to other European countries shown in relevant assessments [6]. Three dairy sheep farms, Farm A, B and C, located in Northern Greece, were used in the case study application. Farms A and B were representatives of the typical semi-intensive farming system as described by Gelasakis et al. [33] and comprised 450 and 272 dairy ewes, respectively. The animals grazed in a common land and were supplemented with concentrate diets and forage feeds (Lucerne Hay and wheat straw); some cereals for the concentrate diets were cultivated on-farm. Farm C comprised 180 dairy ewes, which were kept under intensive farming conditions [33] and fed a concentrate diet together with Lucerne Hay and wheat straw; concentrate and forage feeds were bought.

Each farm was visited by the same veterinarian involved in the development of the system. During the visit, the farmer was asked to provide primary farm management data for the recent production period (year 2018) regarding all the input parameters required by iSAGEDSS in order to create an evaluation scenario (Scenario 1). All input data for Farms A, B and C are provided in Supplement S2 Table S1, Table S2 and Table S3, respectively. Based on these data, the farm income, variable costs and gross margin of each farm were calculated in order to be compared with the predictive outputs of the system. The quality of the predictions was evaluated using the mean absolute percentage error (MAPE) using Equation (7):

(7) $$\mathrm{MAPE}\;=\;\frac{1}{n}\overset{n}{\underset{i\;=\;1}{\sum }}\left|\frac{y_{i}\;-{ŷ}_{i}}{y_{i}}\right|\;\times \;100\%$$

where n = sample size (number of farms); y_i_ = actual values; ŷ_i_ = predicted values.

In Farm A, a second, projectional (what-if) scenario (Scenario 2) for gross margin maximization was created in which inefficient management practices identified from Scenario 1 were altered and an additional source of income by selling 5% of lambs that were initially intended for slaughter was tested. Then, a third, projectional scenario (Scenario 3) was created, simulating the possibility of a future reduction in the price of milk by 5% while incorporating the management changes from Scenario 2.

### 2.7. Ethics Statement

Ethics approval was obtained by the Hellenic Data Protection Authority (ΓΝ/ΕΞ/1473-1/16-06-2016) within the framework of the iSAGE project. Moreover, no data that can be related to or traced to a person’s identity were collected and, in all cases, users tested the system anonymously.

## 3. Results

### 3.1. User Interface

**Figure 2:**
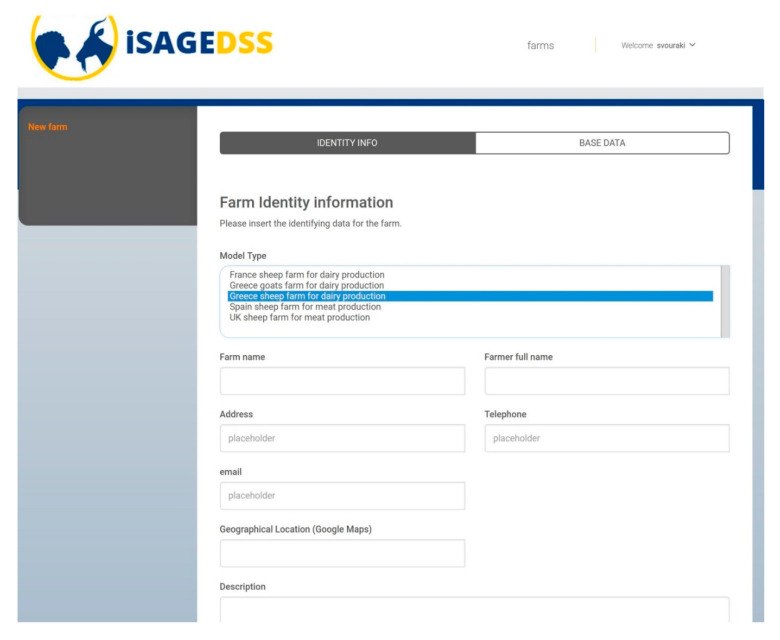
Creation of farm—farm identity information; users choose between five available model types based on production system and country.

**Figure 3:**
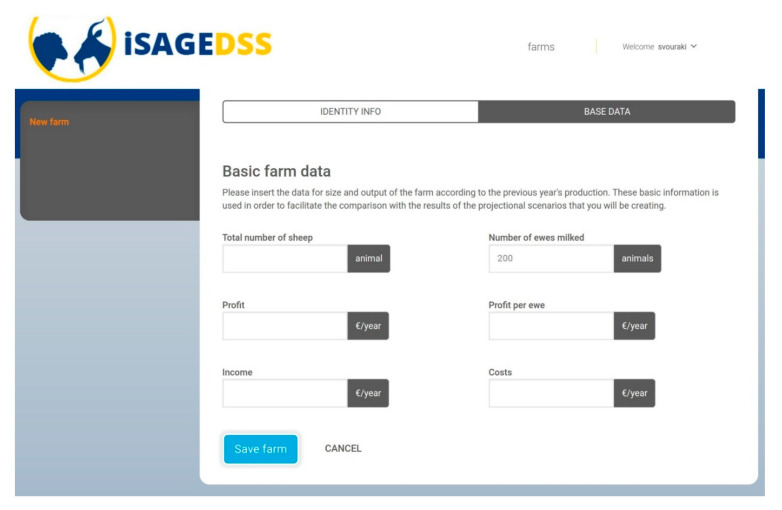
Creation of farm—farm basic data; users provide useful information for the recent production period (costs refer to the variable costs of the farm).

**Figure 4:**
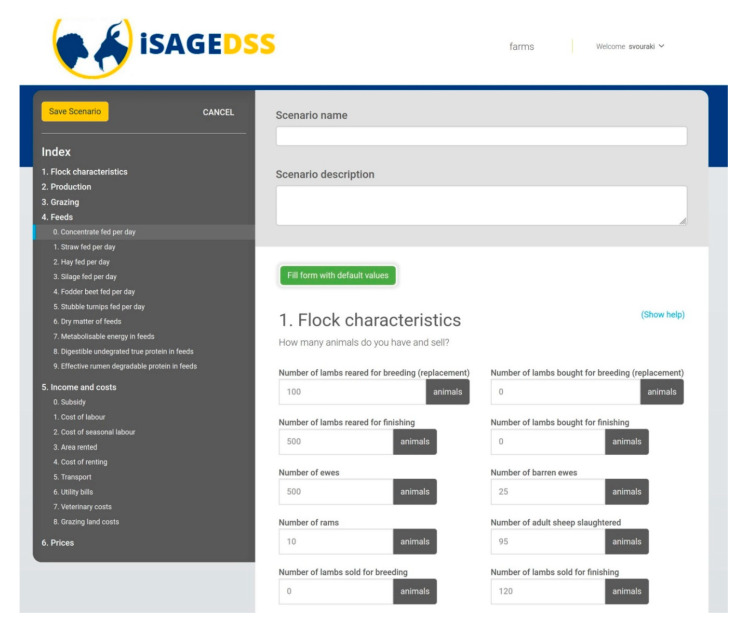
Scenario data input form; users are asked to provide data in terms of flock size, production goals, feeding, grazing, incomes and costs and prices, using a simple web interface.

The iSAGEDSS has a user-friendly interface for small ruminant farmers, which includes the following steps:

1. User login to the iSAGEDSS website: Firstly, users have to access the iSAGEDSS website (www.isage-dss.eu) and login using the unique username and password provided.
2. Create farm: Users accessing the system for the first time have to create their farm by providing some identity information (Figure 2) and some basic data for the recent production period (Figure 3). Data required within the identity information form include contact details of the farmer and the model type (production system and country) to be used. Basic data regarding the recent production period include number of animals and financial figures. These data can be used to compare the outcomes of a future scenario with the baseline situation.
3. Access/Create scenarios: Within their farm, users can have access to their existing scenarios (for editing or viewing results) and they can create new ones.
4. Scenario data input: In their new scenario, users have to input information regarding all important farm aspects; flock characteristics, production, processing (only in dairy sheep and goat farms), grazing, feeds, income and variable costs (without value added tax) and farm prices. Easy navigation through the different categories is provided by the “index option”. Users can use the “Show help” option in order to be provided with further explanations for each of the required data. This option ensures methodological homogeneity for data provision. Moreover, they can also use the “fill form with default values” option to save time in case they wish to test an average management scenario or they are interested in changing only a few parameters (Figure 4).
5. Report page: After all the information has been filled in and the scenario saved, users are navigated to the report page, where they are provided with financial, production and environmental-related figures.

### 3.2. Main Features

#### 3.2.1. Future What-If Scenarios

The iSAGEDSS offers simulations of future scenarios for annual farm management planning. It enables farmers to create and compare many different scenarios by testing all important farm aspects that impact on profitability and productivity in order to establish the most efficient management plan. The expected frequency of use is once per year.

#### 3.2.2. Different Production and Farming Systems Tailored to the Needs of Different Countries

**Figure 5:**
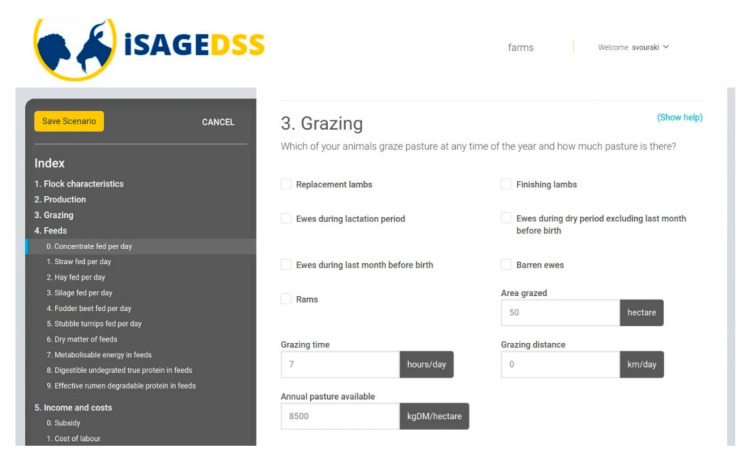
Grazing section of the input parameters page; users input whether their animals will be grazing or not and provide relevant information for grazing.

This decision support system accommodates different production and farming systems and European countries. Specifically, farmers choose the type of model to be used based on the production system and country (Figure 2), and under the grazing section, they input whether their animals will be grazing or not (Figure 5). Moreover, they can use default values specific to their country in cases of input parameters for which they may not have the required information, such as pasture availability or energy and protein in feeds.

#### 3.2.3. Reports on Profitability, Productivity and Environmental Sustainability

**Figure 6:**
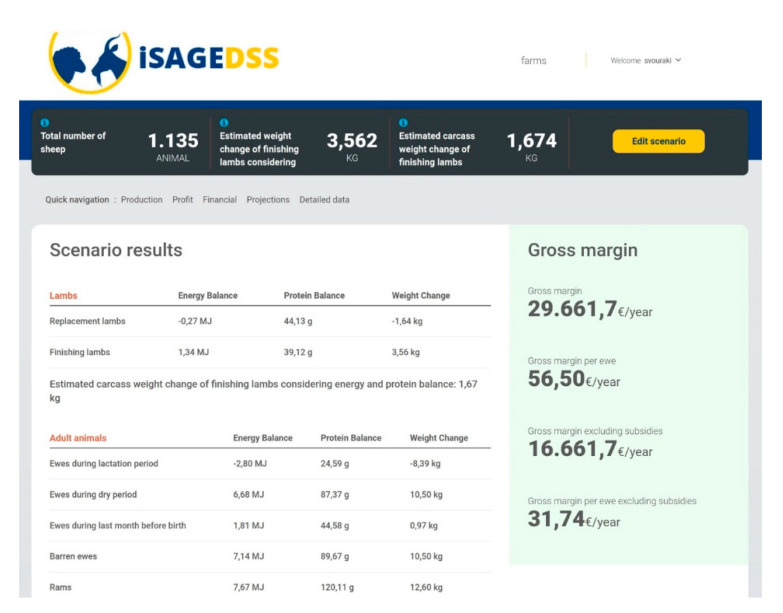
Section of the iSAGEDSS report page with estimated gross margin (with and without subsidies) taking into account production estimates such as live weight and carcass weight change of finishing lambs, based on their energy and protein balance; decimal comma is used as the decimal separator.

**Figure 7:**
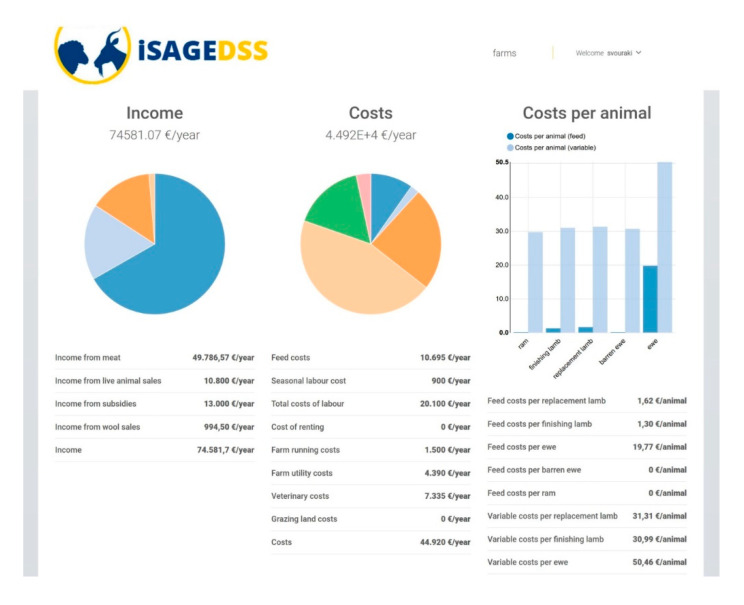
Section of the iSAGEDSS report page with a graphical representation of farm income and costs and costs per animal category; decimal comma is used as the decimal separator.

**Figure 8:**
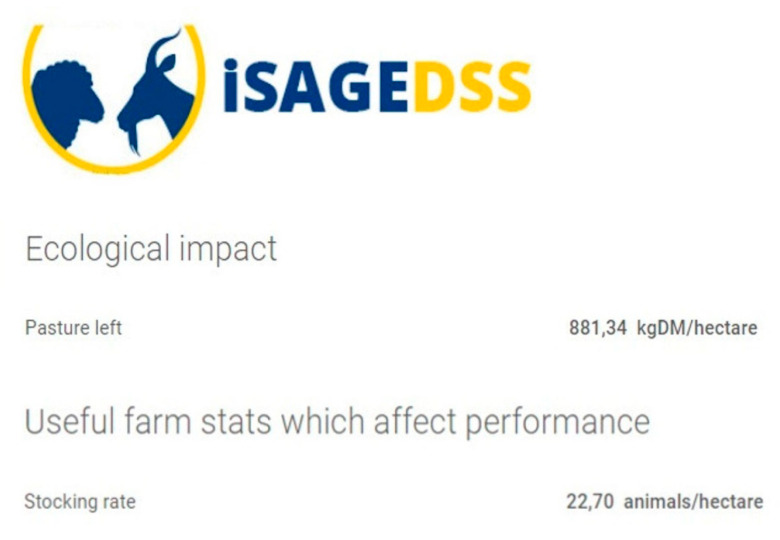
Section of the iSAGEDSS report page with estimated pasture availability at the end of the year and stocking rate; decimal comma is used as the decimal separator.

The users are provided with reports that are mainly focused on profitability and productivity. Specifically, farm income, variable costs and gross margin are estimated taking into account production estimated figures such as live weight and carcass weight of finishing lambs and milk production of lactating animals (according to the production system) based on their nutritional management (Figure 6). Moreover, iSAGEDSS provides comprehensible charts with a breakdown of income and variable costs as well as bar plots portraying the cost of feeding and the total variable costs for each animal category (Figure 7). Finally, for farms where animals graze, pasture availability at the end of the year and stocking rate are estimated, providing useful insight into their environmental sustainability (Figure 8).

### 3.3. Case Study Application Results

The comparison between the case study farms’ actual and iSAGEDSS-estimated annual financial results and ewe milk production are presented in Table 2. No differences were reported in terms of variable costs. However, in all farms, the estimated income was higher compared to the actual one, resulting in a MAPE of circa (ca.) 4%. Consequently, in each case, a higher gross margin was estimated in relation to the actual with a MAPE of ca. 9%. Finally, a milk production increase was estimated compared to the one reported by the farmers, resulting in a MAPE of ca. 5%.

**Figure 9:**
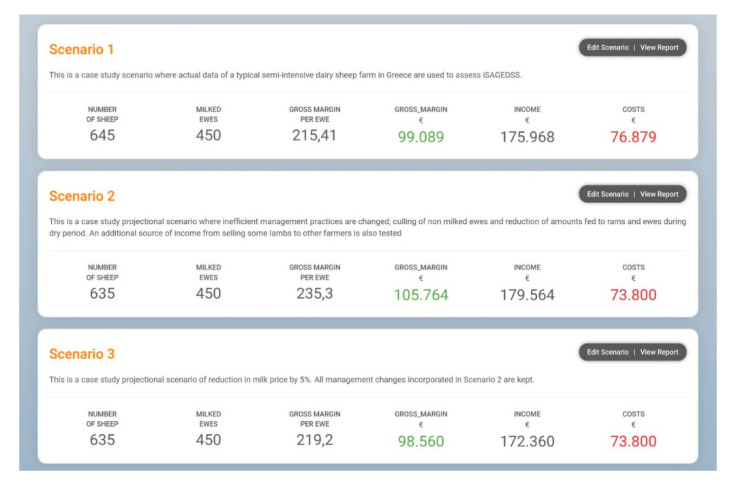
Comparison of the basic financial outputs of Scenarios 1, 2 and 3 from the case study dairy sheep Farm A, in Greece; decimal comma is used as the decimal separator.

Detailed production outputs from Scenario 1 of Farms A, B and C are presented in Supplement S3 Figure S1–S3, respectively. In Farm A, non-milked ewes and rams had a highly positive energy and protein balance. Although to a lesser extent, ewes during the dry period and last month before birth were also fed above their nutritional requirements (Supplement S3 Figure S1). Therefore, in Scenario 2, the amounts of supplemented concentrate feeds for rams and ewes during the dry period and last month before birth were reduced by 50%, 50% and 31%, respectively. Moreover, all non-productive ewes were culled. These practices resulted in a decrease in farm costs by 4.00% compared to Scenario 1 (Figure 9). Moreover, by selling 5% of lambs, the farm income was increased by 2.04% (Figure 9). Consequently, a gross margin increase of 6.74% was achieved (Figure 9). Additionally, a better energy and protein balance was estimated for rams and ewes during the dry period (Supplement S3 Figure S4). Finally, the above management changes resulted in a decrease in the available pasture at the end of the year by 0.45%.

The comparison between the basic outputs of Scenarios 1 and 3 of Farm A (Figure 9) showed that the farm could remain relevantly stable in terms of finances under the possibility of a future reduction in the price of milk (by 5%), by incorporating the management practices tested in Scenario 2. Specifically, the farm’s gross margin was reduced by 0.53% in Scenario 3 compared to Scenario 1.

## 4. Discussion

As asserted in the Introduction, the European sheep and goat sector faces many challenges, which should be properly addressed for a sustainable and competitive future [34,35]. Towards this end, boosting innovations in farm management is considered as one of the main strategic priorities [7,34]. Taking into consideration these issues, we designed and developed iSAGEDSS, a decision support system for sheep and goat farmers that focuses on the efficient management of dairy and meat small ruminant farms in European countries.

The iSAGEDSS is an extension of an existing decision support system, Happy GoatS [17], which was designed based on the dairy sheep and goat sectors in Greece. In iSAGEDSS, adjustments were made to better meet the needs of these sectors; for example, more production-related input parameters were included. Environmental sustainability was also introduced on the basis of efficient grazing management for pasture availability. Moreover, sheep and goat farming across Europe is characterized by a great diversity in terms of production aims and farming systems, resulting in a number of farm types with different needs [29]. In order to deal with this challenge, iSAGEDSS goes beyond Happy GoatS by accommodating diverse production systems and countries. These currently include the meat sheep production system in the UK and Spain, the dairy sheep production system in France and Greece and the dairy goat production system in Greece. This was made feasible by using different sets of default values and acceptable ranges for a range of parameters per system and country provided by relevant research and industry experts involved in the iSAGE project. Such an approach increases the robustness of the results since it allows farmers to use default values specific to their country for any input parameters for which they may not have the required information and protects them from inputting unrealistic data.

Reliable farm-level data have been highlighted as a significant challenge towards the effective design and delivery of decision support systems in agriculture. In order to improve data reliability, automation in data collection through remote sensors has been proposed [27,28]. However, this approach would require prior equipment installations on the farm. Sheep and goat farmers do not easily invest in such equipment, especially given the current financial challenges faced by the sector. Therefore, iSAGEDSS requires farmers to input average data for testing future management decisions, assisted, however, with default values and ranges provided by experts on the field.

Other innovations in the sheep and goat industry have mostly focused on addressing precision feeding, novel feedstuffs [36], genetic improvement of local breeds [37] and electronic identification issues [38]. Regarding farm management, available tools are limited. Most of the existing solutions for sheep and goats have focused on providing sustainability assessments at the farm level [13,15,16]. Specifically, they highlight current sustainability scores in sheep and goat farms with respect to mostly environmental, financial and social indicators. Such tools are operating offline and they require a vast number of input data by the farmer. Other management systems offer historical per-animal recording for various production and health aspects, sometimes combined with precision livestock farming technologies, such as radio frequency identification, for animal tracking and monitoring of diseases [19,23,39]. However, the process of inputting data in such systems is very time consuming and these data have to be constantly updated by the farmers or their consultants. Additionally, in some cases, they can only be fully utilized by farmers that are members of breeding programs and milk controlling schemes [23]. Finally, there are some other tools which aim only at dealing with specific problems such as greenhouse gases, biodiversity and water [18] feed formulation [14], control of parasites [21] and GPS-enabled animal tracking [40].

Contrary to all the above, iSAGEDSS offers projectional capabilities for decision-making with an emphasis on profitability and productivity, while considering grazing management. To the best of our knowledge, only PASTOR-DSS, a decision support system for dairy sheep farming, allows for bio-economic simulations. However, since simulations rely on an individual animal models, its use is impaired by the large number of required input animal parameters [22]. Although iSAGEDSS takes into account all important farm aspects, great effort was put into limiting the number of input variables to those absolutely required and, therefore, the time investment for the farmer; the input data form can be completed in approximately 30 min and the report is generated in seconds. Moreover, since it is designed for annual farm management planning, it has a low frequency of use, with expected updates once per year, ideally prior to the next production period. At the same time, the fact that it is an online (cloud-based) web application with a user-friendly interface and visual presentation of results further differentiates iSAGEDSS from most of the existing sheep and goat management tools and can help towards increasing adoption rates [24,25].

In terms of accuracy, the presented case study in three dairy sheep farms (two semi-intensive and one intensive) in Greece, where actual data were compared with the estimated ones, showed a MAPE of 9.04% and 4.84% for gross margin and milk production estimation, respectively. According to Lewis [41], values below 10% are considered as highly accurate forecasting. The error concerning gross margin was a result only of income estimation; no differences were reported regarding variable costs. The latter was expected since the estimation of variable costs is based solely on the input data. On the other hand, income estimation takes into account the production estimates—in this case, the milk production change (compared to that inputted) as a result of nutritional management. The calculated error concerning milk production could be related to the default values used for metabolizable energy and protein in feeds. The energy and protein in feeds can fluctuate between different regions of a country depending on the agronomic quality of the land as well as between different batches of the same land. Therefore, if chemical composition analyses of the used feedstuffs were available, even more accurate estimations could be expected.

In terms of impacts, three dimensions can be acknowledged based on the system’s available features. Firstly, iSAGEDSS helps sheep and goat farmers in different European countries to make annual management planning decisions by simulating future scenarios. There are a number of scenarios that could be potentially simulated such as flock size optimization, production optimization, pricing fluctuations, pasture availability fluctuations, extensification/intensification of the production system and different feeding strategies. By creating many different scenarios, farmers could understand the impact that each management decision may have on their profitability and establish an efficient plan for managing their farm. Therefore, iSAGEDSS can help towards increasing the currently low professionalization of the sector [1,3].

Moreover, it supports production optimization and gross margin maximization while also reducing dependence on public subsidies, which have been described as major challenges for the sheep and goat industry [1,5,42]. Specifically, production-related figures are estimated based on energy and protein balance, which are then taken into account in the financial results. The latter include a total estimation and a breakdown of income and variable costs as well as gross margin with and without the inclusion of direct and coupled subsidies and compensations. This way, farmers may identify their most important expenses, the cost of maintaining non-productive animals in their business, the extent to which their income is dependent on public support and potential alternative sources or solutions that could increase their profitability and productivity. Finally, it stimulates environmental awareness by allowing farmers to take into account the amount of annual pasture available and by estimating the pasture availability at the end of the year. Of course, a certain level of caution needs to be exercised when interpreting such estimates since weather conditions can affect pasture availability. Given the deterministic nature of the model, randomness is not incorporated in its estimation approach. However, users can simulate different scenarios of annual pasture availability under the hypothesis of adverse weather conditions in order to create an efficient grazing management plan. Efficient grazing management is expected to help farmers towards adjusting to new environmental policies [6].

Such impacts were demonstrated in the presented case study application in Farm A by simulating a scenario of alternative management practices, compared to the farm’s current ones, for gross margin maximization and a scenario of milk pricing fluctuation. According to our experience, these are the most common cases of decision-making that farmers are interested in. Moreover, in the past few years, Greek sheep farmers have indeed been faced with reduced milk prices enforced by dairy companies. To test the above, Farm A was chosen as the best representative on the basis of having the most accurate predictions. Based on our results, the gross margin of the studied farm could be increased by ca. 7% by culling all the non-productive ewes, incorporating a more efficient nutritional management strategy and utilizing a new source of income through the selling of lambs as replacements to other farmers. Moreover, changes in nutritional management were feasible without a considerable decrease in pasture availability at the end of the year (ca. 0.5%). Additionally, given all the above management changes, a possible reduction in the price of milk by 5% was found to result in an insubstantial gross margin reduction compared to the farm’s current situation (ca. 0.5%). Such findings confirm that iSAGEDSS can help farmers to plan for efficient and sustainable farm management even under adverse hypothetical scenarios.

All the above features and impacts of iSAGEDSS suggest that it has the potential to be easily adopted by farmers. Specifically, according to studies and applications in agriculture, a holistic approach for management solutions, user-friendliness and cloud-based services for greater availability and applications [8,9] are key elements for addressing implementation problems and increasing adoption rates of decision support systems for farm management [24,25]. Moreover, a desirable performance in terms of prediction accuracy and provision of decision support for higher productivity and profitability has been reported as a core factor for adoption [25]. Finally, compliance with legislative demands, such as the new environmental policies for grazing management, has been suggested as a driving factor that could further motivate farmers in the uptake of relevant tools [25]. Ease of adoption is further strengthened by the involvement of end-users (farmers) [43] as well as consultants and research actors in the development of iSAGEDSS.

Our main goals for the future are to further evaluate the ease of adoption of iSAGEDSS through an extensive case study/survey with farmers and to achieve the long-term utility of the decision support system. Towards the latter, a team of sheep and goat experts will be available for farmers to communicate with if they require consultancy on their results and would like to discuss more viable solutions. Moreover, given the fact that not all farmers have a good knowledge of managing applications and online products based on their educational status [26] and are not familiar with the English language, we intend to include step-by-step instructions for using iSAGEDSS in its hosting website and incorporate translation features. At the same time, ongoing support will be provided by the development company for the maintenance of the website page, the protection of users’ personal accounts and the monitoring of the system in order to make sure that any technical problems are quickly dealt with. Training, consultancy and provision of automated or semi-automated technical assistance have been acknowledged as important incentives for the adoption of decision support systems by farmers [26,44]. Moreover, the possibility of including new variables associated with heterogeneity factors, such as the breed, and estimating more environmental-related figures, such as greenhouse gas emissions, will be explored and any adjustments needed in order to better meet the needs of the market will be incorporated accordingly. In the case of newer versions, special emphasis will be placed on retaining existing users’ data and simulated scenarios. Finally, iSAGEDSS aspires towards future potential growth in other interested countries given that default values and acceptable ranges will be provided by relevant experts.

## 5. Conclusions

The iSAGEDSS is a web-based, model-driven decision support system which focuses on the efficient management of meat and dairy small ruminant farms. It was developed as a follow-up to the Happy Goats system. iSAGEDSS allows sheep and goat farmers of different production and farming systems in different European countries (UK, Spain, France and Greece) to test future annual management decisions by estimating both financial and production-related figures with a high prediction accuracy. Environmental figures relating to grazing management are also estimated. All these are illustrated with simple and comprehensible charts and plots. In this regard, iSAGEDSS is expected to help small ruminant farmers to understand the impact of their management decisions and increase their professionalization with the aim of responding better to the challenges faced by the sector and remaining sustainable.

## Figures and Tables

**Table 1:** Data provision for default values and acceptable ranges per production system and country.

Production System	Country	Data Provision ^1^
Meat sheep	United Kingdom	AHDB & NSA
Meat sheep	Spain	Oviaragón
Dairy sheep	France	IDELE
Dairy sheep	Greece	AUTH
Dairy goats	Greece	AUTH

^1^ AHDB = Agriculture and Horticulture Development Board; NSA = National Sheep Association; Oviaragón = Oviaragón Pastores Grupo Cooperativo; IDELE = Institut de l’elevage; AUTH = Aristotle University of Thessaloniki (Laboratory of Animal Husbandry).

**Table 2:** Comparison between actual (year 2018) and estimated annual financial data and ewe milk production in the case study of dairy sheep farms (Farm A, B and C) in Greece.

	Farm	Actual Data	Estimated Data	MAPE (%) ^1^
Gross margin (euros/year)	A	95,593	99,089	9.04
	B	72,111	78,591	
	C	21,102	24,157	
Income (euros/year)	A	172,472	175,968	3.47
	B	145,400	151,880	
	C	78,047	81,103	
Variable costs (euros/year)	A	76,879	76,879	0.00
	B	73,289	73,289	
	C	56,945	56,945	
Milk production (kg/ewe/year)	A	311	319.1	4.84
	B	370	392.3	
	C	300	317.7	

^1^ MAPE = mean absolute percentage error.

## Supplementary Materials

The following are available online at https://www.mdpi.com/2076-2615/10/12/2421/s1, Supplement S1 Table S1: Input data, default values and acceptable ranges for meat sheep production system in the United Kingdom, Supplement S1 Table S2: Input data, default values and acceptable ranges for meat sheep production system in Spain, Supplement S1 Table S3: Input data, default values and acceptable ranges for dairy sheep production system in France, Supplement S1 Table S4: Input data, default values and acceptable ranges for dairy sheep production system in Greece, Supplement 1 Table S5: Input data, default values and acceptable ranges for dairy goat production system in Greece; Supplement S2 Table S1: Input data used in Scenario 1 (validation scenario with actual data from 2019 production) of the case study in dairy sheep Farm A in Greece, Supplement S2 Table S2: Input data used in Scenario 1 (validation scenario with actual data from 2019 production) of the case study in dairy sheep Farm B in Greece, Supplement S2 Table S3: Input data used in Scenario 1 (validation scenario with actual data from 2019 production) of the case study in dairy sheep Farm C in Greece; Supplement S3 Figure S1: Production related outputs (energy and protein balance and weight change) of different animal categories as estimated in Scenario 1 of the case study application in Farm A, Supplement S3 Figure S2: Production related outputs (energy and protein balance and weight change) of different animal categories as estimated in Scenario 1 of the case study application in Farm B, Supplement S3 Figure S3: Production related outputs (energy and protein balance and weight change) of different animal categories as estimated in Scenario 1 of the case study application in Farm C, Supplement S3 Figure S4: Production related outputs (energy and protein balance and weight change) of different animal categories as estimated in Scenario 2 of the case study application in Farm A.
